# Faculty perceptions, awareness and use of open educational resources for teaching and learning in higher education: a cross-comparative analysis

**DOI:** 10.1186/s41039-022-00185-z

**Published:** 2022-03-25

**Authors:** Victoria I. Marín, Olaf Zawacki-Richter, Cengiz H. Aydin, Svenja Bedenlier, Melissa Bond, Aras Bozkurt, Dianne Conrad, Insung Jung, Yasar Kondakci, Paul Prinsloo, Jennifer Roberts, George Veletsianos, Junhong Xiao, Jingjing Zhang

**Affiliations:** 1 Carl von Ossietzky University of Oldenburg, Oldenburg, Germany; 2 Present Address: University of Lleida, Lleida, Spain; 3 Anadolu University, Eskisehir, Turkey; 4 Innovation in Learning Institute, University of Erlangen-Nuremberg, Erlangen, Germany; 5 University of South Australia, Adelaide, Australia; 6 Athabasca University, Athabasca, Canada; 7 International Christian University, Mitaka, Japan; 8 Middle East Technical University, Ankara, Turkey; 9 University of South Africa, Pretoria, South Africa; 10 Royal Roads University, Victoria, Canada; 11 Shantou Radio and Television University, Shantou, China; 12 Beijing Normal University, Beijing, China

**Keywords:** Open educational resources (OER), Repositories, Higher education (HE), Faculty members, International comparison

## Abstract

This paper explores faculty’s perspectives and use of open educational resources (OER) and their repositories across different countries by conducting a multiple case study to find similarities and differences between academics’ awareness, perceptions and use of OER, as well as examining related aspects of institutional policy and quality that may influence individual views. Data were collected through nine expert reports on each country studied (Australia, Canada, China, Germany, Japan, South Africa, South Korea, Spain and Turkey) and were analyzed through qualitative content analysis using thematic coding. Findings show the impact on individual OER adoption with regard to the individual control of diverse factors by faculty members; of institutional policies and quality measures on the externally determined factors (by the institution); and of institutional professional development and provision of incentives in more internally determined factors (by the faculty members themselves). These findings carry implications for higher education institutions around the world in their attempt to boost OER adoption by faculty members.

## Introduction

Open educational resources (OER) have yet to be widely adopted in higher education (HE), despite their affordances for education and increasing interest from the educational community (Bozkurt et al., 2019; Murphy, 2013). This situation is caused in part by macro-level factors such as national regulations, funding possibilities and existing OER infrastructure; meso-level factors such as institutional policies, OER promotion measures and specific infrastructures (Conole, 2012; Marín et al., 2020a; under review; Yuan et al., 2008); and micro-level factors such as faculty perceptions, awareness and use of OER in teaching and learning (Cox & Trotter, 2017), which in turn are affected by macro- and meso-level factors. Factors at the three levels are considered to be interdependent (Marín et al., 2020b; Zawacki-Richter, 2009).

Prior research focusing on factors influencing OER adoption by individual faculty members has found, for example, appropriate institutional support to be an enabler, while inadequate support hinders OER practice (Baas et al., 2019; Bates et al., 2007; Belikov & Bodily, 2016; Bossu et al., 2014; Schuwer & Janssen, 2018). Institutional factors also include institutional readiness as OER creators, institutional culture and volition (Cox & Trotter, 2017), institutional reputation (Bates et al., 2007; Rolfe, 2012), cost benefit (Bates et al., 2007; Belikov & Bodily, 2016), pedagogical benefits (Belikov & Bodily, 2016; Schuwer & Janssen, 2018) and availability of quality OER (Baas et al., 2019; Bossu et al., 2014). The adoption of OER is also influenced by individual faculty factors, for example, when practitioners are not equipped with adequate knowledge and/or skills required for OER (Belikov & Bodily, 2016; Cox & Trotter, 2017; Li & Li, 2012). Individual awareness of OER affordances is found to be a barrier to implementation (Baas et al., 2019; Bates et al., 2007; Li & Li, 2012; Reed, 2012; Rolfe, 2012; Schuwer & Janssen, 2018), whereas adequate awareness contributes to OER practice (Mtebe & Raisamo, 2014) although Bossu et al. (2014) showed that awareness was not positively correlated with adoption or creation. Both individual reputation and time availability are also factors influencing individual OER adoption (Bates et al., 2007; Rolfe, 2012; Schuwer & Janssen, 2018). Finally, research findings included some macro-level factors that also impact on individual OER adoption, for example, intellectual property policies (Bates et al., 2007; Bossu et al., 2014).

Despite these findings, it is worth noting that only a few studies have based their work on a conceptual framework (e.g., Baas et al., 2019; Cox & Trotter, 2017; Mtebe & Raisamo, 2014), looked comparatively at these aspects across countries (e.g., Jung & Lee, 2020) or/and used multiple data sources (e.g., Baas et al., 2019; Bossu et al., 2014). This study is significant in that the application of a conceptual framework is intended, not only to provide contextual information that helps to explain and interpret data, but also to contribute to the further development and revision of the framework. Its uniqueness also lies in a much wider-scale comparison involving more institutions across more countries. Findings from this study may be more generalizable. This is especially important, considering the increased need for digital accessible educational resources due to COVID-19 (Huang et al., 2020). In addition, this study has an exclusive but more comprehensive focus: It is exclusive in that it concentrates on micro-level OER practice, but more comprehensive in that it covers all key aspects of OER practice by faculty. In this sense, aspects related to OER infrastructure, quality, policy and promotion of change have not been well addressed comparatively in the literature so far and are worthy of a careful investigation to increase a general comprehensive understanding of OER factors influencing individual OER adoption worldwide and, ultimately, to aid in the search for collective, global and particular, solutions to support this adoption.

As part of the research project “Digital educational architectures: Open learning resources in distributed learning infrastructures - EduArc” (https://uol.de/coer/research-projects/projects/eduarc), and as a follow-up to the macro- and meso-level studies (i.e., national systems and institutional infrastructures and organization (see Marín et al., 2020a; under review), this study aimed to analyze aspects related to the use, creation, remix and sharing of OER in HE at the micro-level of teaching and learning (that is, faculty members’ experiences) across countries. The use of the term OER in this study does not exclude our recognition that fully open educational resources are not always possible depending on institutional HE policies. The framework of content analysis for the macro- and meso-level studies was adopted, with the unique features of the micro-level also taken into account, namely infrastructure (local environment), quality (quality of OER), policy (local policies) and change (incentives and faculty support).

Against this background, the research questions are as follows:

- *RQ1* What are faculty members’ perceptions and use of infrastructure (e.g., tools and platforms) and types of OER across countries?
- *RQ2* How aware are faculty members of OER quality assurance (QA) institutional procedures and who oversees them across countries?
- *RQ3* To what extent are faculty members familiar with institutional OER policies and the possibilities to get involved in institutional OER policymaking across countries?
- *RQ4* How are faculty members motivated to use OER in their teaching practices across countries?

### Conceptual framework of study

Theories and models have been developed over time to explain why individuals adopt particular technologies. Well-known models are, for instance, the *Technology Acceptance Model* (TAM) for analyzing the acceptance and usage of (an specific) technology, or the *Unified Theory of Acceptance and Use of Technology* (UTAUT) that focuses on the impact on behavioral intention of various determinants (Venkatesh et al., 2003) and has been used in OER adoption studies in HE (e.g., Mtebe & Raisamo, 2014). Its enhanced version (UTAUT2) includes three individual determinants in technology adoption that are moderated by age, gender and experience (Venkatesh et al., 2012). In a cross-cultural study on the adoption of OER in HE (Jung & Lee, 2020), the authors added *culture* to the UTAUT2 model as an important moderating variable and incorporated two cross-cultural frameworks: Hall’s (1976) high-context and low-context cultural theory and Hofstede’s (2001) cultural values framework.

Despite their interesting insights into OER adoption, these models neither specifically address its complexity nor the broader scope of research required. For example, Mtebe and Raisamo (2014) identified other factors different from the ones considered by UTAUT that are influencing OER adoption by faculty. A comprehensive model that has been used in some studies exists in the context of OER adoption by faculty: the *OER Adoption Pyramid* (Cox & Trotter, 2017).

**Fig. 1:**
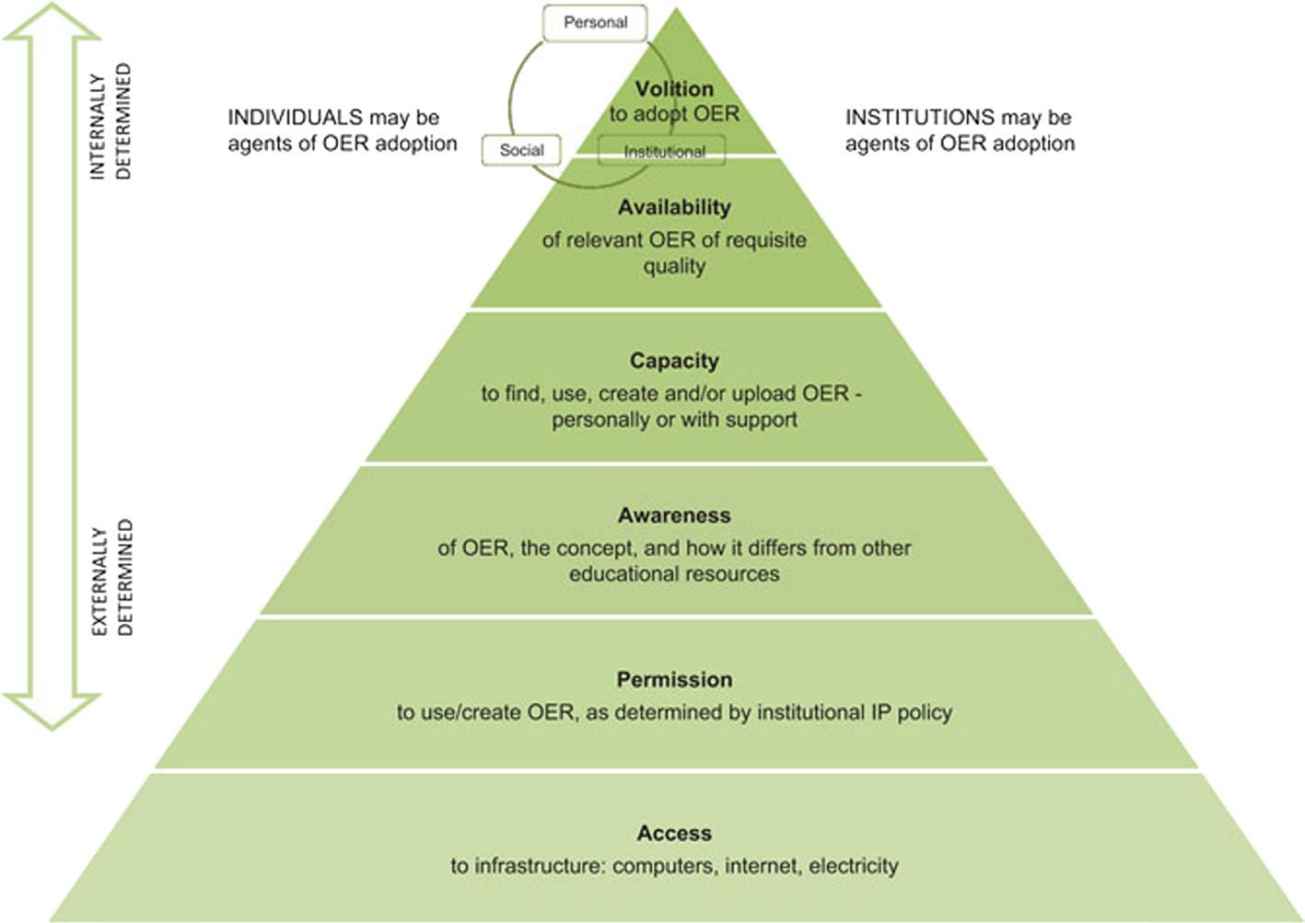
OER adoption pyramid. *Note* From “An OER framework, heuristic and lens: Tools for understanding lecturers’ adoption of OER” by G. Cox and H. Trotter, 2017, *Open Praxis, 9*(2), p. 155. (https://doi.org/10.5944/openpraxis.9.2.571)

The framework (see Fig. 1) considers different layers moving from externally determined (national, province or institutional level) to internally determined (individual level) factors, with each of them needed to support the next layer above. The first layer addresses access to infrastructure; the second one refers to permission to use/create OER; the third layer is related to awareness of OER, what it involves, and how it differs from other educational resources; the fourth layer addresses capacity, i.e., the skills to find, use, create and/or upload OER; the fifth layer is about availability of relevant OER of quality; and the last layer concerns volition to adopt OER, which includes individual, social, and institutional volition. According to the context in the study by Baas et al. (2019), availability should be considered a prerequisite for instructors to explore their capacity and volition and, therefore, be lower in the pyramid. However, considering different contexts in the countries involved in this study, the original version of the model has been taken into account. The OER Adoption Pyramid was used in this study to facilitate qualitative data analysis and generate new insights that lead to an enhanced model.

## Methodology

### Aim, design and sample

Our study is based on an interpretative paradigm and aims at identifying and understanding factors influencing individual adoption of OER in HE across countries. To this end, we conducted a comparative multi-case study (Yin, 2009), consisting of nine cases based on written reports collected from members of the Center for Open Education Research (COER) (https://uol.de/coer) from nine countries as a convenience sample: Australia, Canada, China, Germany, Japan, South Africa, South Korea, Spain and Turkey.

**Fig. 2:**
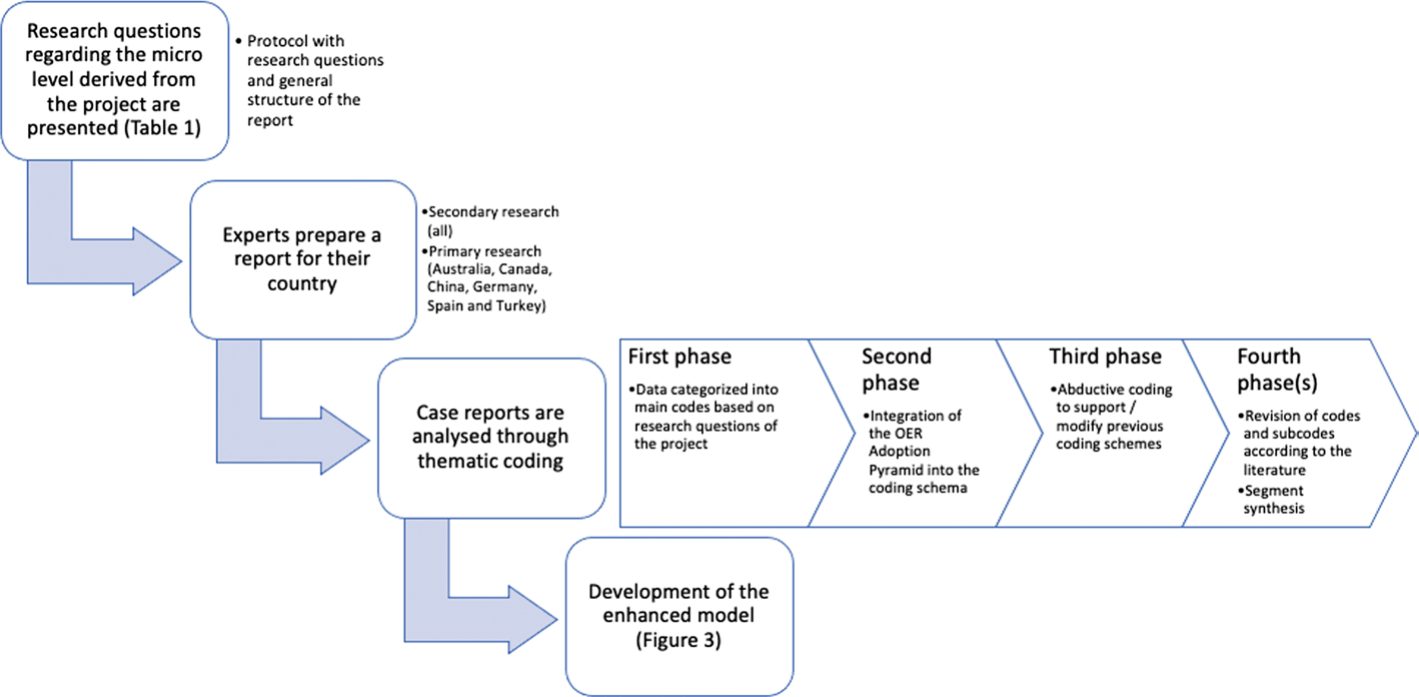
Design process of the study

The whole design process is depicted in Fig. 2 and described below.

In the first phase, common research questions for the country reports were provided based on the consideration of four main elements: infrastructure, quality, policy and measures for change (see Table 1). These research questions were based on the consideration of the three broad meta-levels of distance education research (Zawacki-Richter, 2009). The microlevel (teaching and learning in distance education) was taken into account and adapted to the situation of OER in any form of education modalities in higher education, considering the particular focus on aspects influencing OER infrastructures of the EduArc project.

**Table 1:** Common research questions to guide the content of the microlevel reports

Infrastructure	Quality	Policy	Change
How do teachers know about and use the existing local infrastructures? Which infrastructures / working environments (e.g., tools, platforms) do teachers prefer to use to create and edit (O)ER? Which types of (O)ER do teachers prefer to use in their teaching? Which functionalities would be helpful for teachers to edit their own or others' (O)ER and/or for collaborative work?	Which aspects do teachers use to define the quality of (O)ER and their infrastructures? Are teachers involved in defining quality of (O)ER and their infrastructures? Are teachers aware of how institutional quality procedures related to (O)ER work and who is in charge of them?	Are there policies specific to certain study programs or departments or schools? Are teachers involved in policymaking? Are teachers aware of institutional policies related to (O)ER?	How are teachers involved in the technical and informational aspect of creating (O)ER and advancing the infrastructures? Are teachers being supported in the technical-informational aspects of (O)ER material creation? How? (e.g., incentives, support) How do teachers integrate external (O)ER into their own (O)ER? What changes do teachers make to their own and external (O)ER? With whom, where and how do teachers share (O)ER?

### Data collection

While much research around OER relies on one or two data sources, this study draws from multiple and complementary data sources contained in each case report, in order to enable a more nuanced analysis of the topic under investigation.

The case reports were mainly based on desk research, i.e., secondary research, such as empirical studies covering local issues on OER, and document analysis (e.g., white papers, policy papers, institutional reports), but some of them included data collected through primary research too, namely: a survey and personal interviews (phase 2 in Fig. 2, for details, see Table 2). As different countries were involved and the situations greatly differ across countries, the autonomy was given to the experts to decide what kind of data were needed to tell coherent stories of their country cases. As an illustrative example, in China’s case, after analyzing the secondary data, experts found that they needed to interview key people who were involved in designing and implementing OERs in higher education institutions to explain how and why things were going as described in the secondary data.

**Table 2:** Data collection instruments used in the country reports

Country	Data collected from			
	Desk research	Analysis of documents	Survey	Personal interview
Australia	X	X	X (*n* = 70)	
Canada	X	X		X (*n* = 8)
China	X	X		X (*n* = 3)
Germany	X	X	X (*n* = 76)	
Japan	X	X		
South Africa	X	X		
South Korea	X	X		
Spain	X	X	X (*n* = 576)	
Turkey	X	X		X (*n* = 5)

With regard to the primary research, personal interviews were designed in a semi-structured form, following the research questions for the micro-level of the corresponding work package of the project. The number of interviews and survey participants predominantly included faculty; however, in a few cases administrators (Turkey) and librarian staff (Australia and Canada) also participated. In the case of China, only administrators were interviewed. Participants also came from different HE institutions; Australian participants were represented by 22 HE institutions, the German survey addressed faculty from HE institutions in the federal state of Lower Saxony, and Spanish participants came from 64 universities and formed a representative sample. The variation in participant numbers—especially in the three quantitative surveys—needs to be kept in mind when results are reported so as to not misinterpret percentages that are provided. All study participants involved had given their informed consent to participate.

By including multiple types of data sources in their country reports (e.g., interviews, surveys, empirical papers, reports, etc.), the experts aimed to add to the qualitative rigor of the study (Thomas & Magilvy, 2011). Data collected by different methods helped increase the richness of the case studies, especially in the cases where desk research was not enough to provide a proper answer to the posed research questions.

### Data analysis

Quantitative and qualitative data from the case reports were analyzed through thematic coding with MAXQDA2020 in several iterations (Miles et al., 2013). MAXQDA2020 is a software for qualitative and mixed methods that can be used for any type of qualitative research and integrates a comprehensive set of tools for collecting and organizing data, analyzing and visualizing data. Each case report was uploaded to MAXQDA2020 as documents for the iterative coding process (third phase in Fig. 2).

In the first iteration, the data were categorized into main codes based on the four elements of the reports described in the research questions (Infrastructure, Policy, Quality and Change). In the second iteration, the OER Adoption Pyramid (Cox & Trotter, 2017) was integrated as a way of understanding some of the elements, especially concerning awareness, capacity, availability and volition. In the third phase of coding, codes and subcodes were added by abductive coding, based on the identification of new topics that were not covered in the two previous deductive phases, considering how the data could support the previous coding schema and call for its modification (Kennedy & Thornberg, 2018). The fourth and final phase of coding involved the revision of some codes and subcodes according to the literature that has explored faculty’s perceptions about OER (e.g., Baas et al., 2019; Belikov & Bodily, 2016; Cox & Trotter, 2017), as well as renaming codes for greater accuracy and deleting redundant codes. In addition, each of the coded segments was given a “comment” in MAXQDA, in order to summarize its content (“the preview”), given that many of them were overly long. This action was carried out in order to facilitate the generation of visualizations of codes–subcodes–segments with MAXMaps (functionality within MAXQDA2020) after the analysis. Each report could have more than one coded segment related to one, more or the same (sub)code. The iterative coding process was conducted by one coder, but the research group reached a consensus regarding the final versions of the codes. The whole coding process took three intensive months, with longer periods concentrated within the third and fourth phases due to their complexity.

**Fig. 3:**
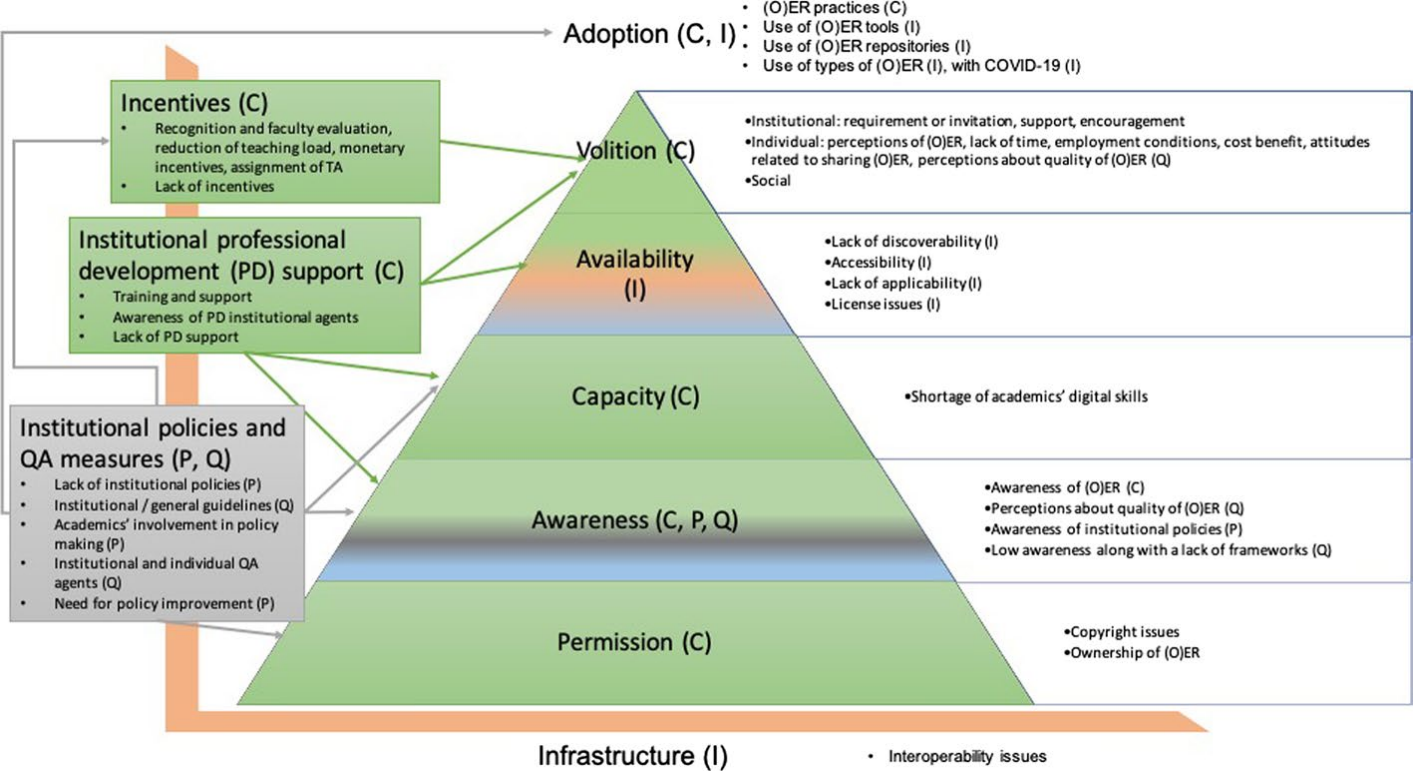
Enhanced OER adoption pyramid combined with infrastructure (I), policy (P), quality (Q) and change (C). *Note* The differentiation between the four aspects is done using parenthesis and colors

This process resulted in an enhanced model that combines the OER Adoption Pyramid and the four elements of the research project at the micro-level based on data from the international reports. Therefore, the enhanced model provides a broader view to the previous literature, which focused exclusively on one institution or country (see Fig. 3, with various codes and subcodes depicted).

### Limitations

This study’s methodological approach presents some limitations that must be acknowledged. As each country expert (or group of experts) was able to organize their case as they wanted, according to the project’s main research questions, we could not report on homogeneous data collection methods as the basis for the reports. Similarly, the primary empirical data do not come from the application of the same instruments; even in the case of using the same data collection method (survey or interview), neither refers to comparative data in terms of the number of participants. Furthermore, although consensus on the final version of the coding schema was reached by the research group, the data analysis involved only one coder and this fact could involve a bias in the interpretation and saturation of the report data. Even though these limitations may make the comparison across countries difficult, the exploration of the same research questions still presents valuable insights into the topic.

## Findings and discussion

### Research question 1: Use and perceptions of OER

This research question considered faculty members’ use of OER infrastructure, as well as types of OER, alongside their perceptions with regard to the challenges of this infrastructure.

#### Faculty use of OER infrastructure and OER types

**Fig. 4:**
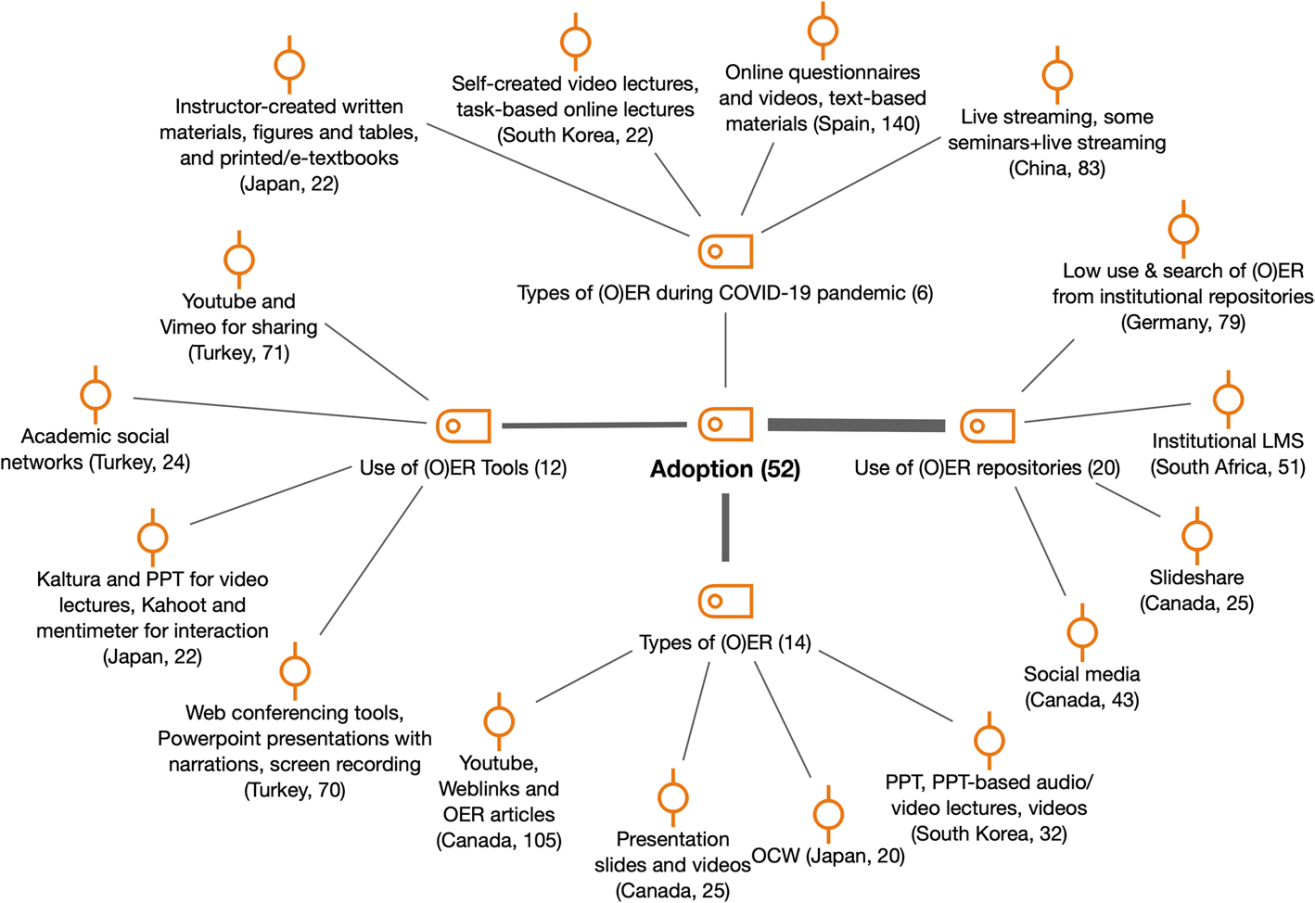
Subcodes and examples for adoption (RQ1). *Note* Umbrella code (i.e., Adoption) is in the center and subcodes (e.g., Use of OER tools) are connected to it. The numbers between parentheses in codes and subcodes (also onwards on in *n* =) refer to the number of coded segments. Examples of those coded segments have been included for each subcode, where the information in parentheses refers to the country report and the line number where that segment is located in the corresponding document. This note applies to the next figures too

Under the umbrella code *Adoption*, we included different subcodes that refer to the appropriation of the OER infrastructure by faculty members (see Fig. 4).

The *use of OER repositories* (*n* = 20) varied in each country. In Canada, one of the interviewees (respondent B) highlighted eCampus Ontario as a “fairly good source of resources”. Also, some interviewees used social media for evidence of new and relevant materials (respondents B and D). In Japan, in the study by Jung et al. (2013) that involved 27 educators, faculty used YouTube as educational content (53.3%), but none of the Japanese faculty members had created video lectures and uploaded them to YouTube. In Spain, faculty members reported using institutional repositories in different ways, but the common ones were as a place to store (and share) OER (especially the institutional virtual learning platform). For instance, one participant in the Spanish survey stated that “(I use OER repositories) to store all the class materials and activities.” However, a high percentage of the Spanish and German faculty participants in the survey studies did not know about the existence of OER repositories in their institutions (27.4% and 36.8%, respectively). A general lack of knowledge about tools and repositories was also identified in Australia in its survey study, in contrast with the research by Bossu et al. (2014). Lack of awareness of OER repositories is an acknowledged barrier to OER access and sharing (Bates et al., 2007).

Concerning *types of OER* (*n* = 14), certain types of resources were common across the countries, especially videos and presentations. For example, in Canada, Hayman’s study (2018) reported on OER use in Ontario with 383 post-secondary educators who participated. The data showed that 79% of participants used YouTube videos, 83% used web links, and 55% used open access articles. In Spain, the most popular OER formats reported by participants in the survey were slide presentations (87.7%), OER in text format (74.5%) and pictures (65.9%), but videos (48.4%) and assessment tests (43.3%) received a high degree of use too. Australian educators preferred to use OER that require little modification, for example, freely available videos such as TedX talks and YouTube clips (Kandlbinder & Chelliah, 2015). In the study by Li (2015) with Chinese academics from the Northwest Normal University, interviewees used mostly images (92%) and audio recordings (69%). These findings are in line with previous literature that shows that instructors are more commonly using technology in teacher-centered approaches than in student-centered ways; therefore, teaching practices are not profoundly transformed (Bond et al., 2018; Blin & Munro, 2008; Marcelo & Yot-Domínguez, 2018; Marcelo-García et al., 2015). This situation has persisted during the COVID-19 pandemic, as most of the *types of OER used by faculty in this period* (*n* = 6) show. For instance, in China, and in particular at Peking University, academics mostly adopted live-streaming, accounting for 50% of the total number of courses (Gong, 2020). Similarly, in a survey of 716 faculty members at Seoul National University in South Korea, over 32% of academics used self-created video lectures, and over 22% offered task-based online lectures (Park, 2020).

#### Challenges regarding OER infrastructure according to faculty perceptions

*Interoperability issues* (n = 3) were among the challenges of OER infrastructure identified by faculty in Turkey, Canada and Spain, also common in the literature (Yuan et al., 2008). For instance, in Spain, 45.6% of survey participants stated that the integration between OER repositories and other institutional systems existed, but a high number of academic staff were unsure of the existence of this integration (34%).

**Fig. 5:**
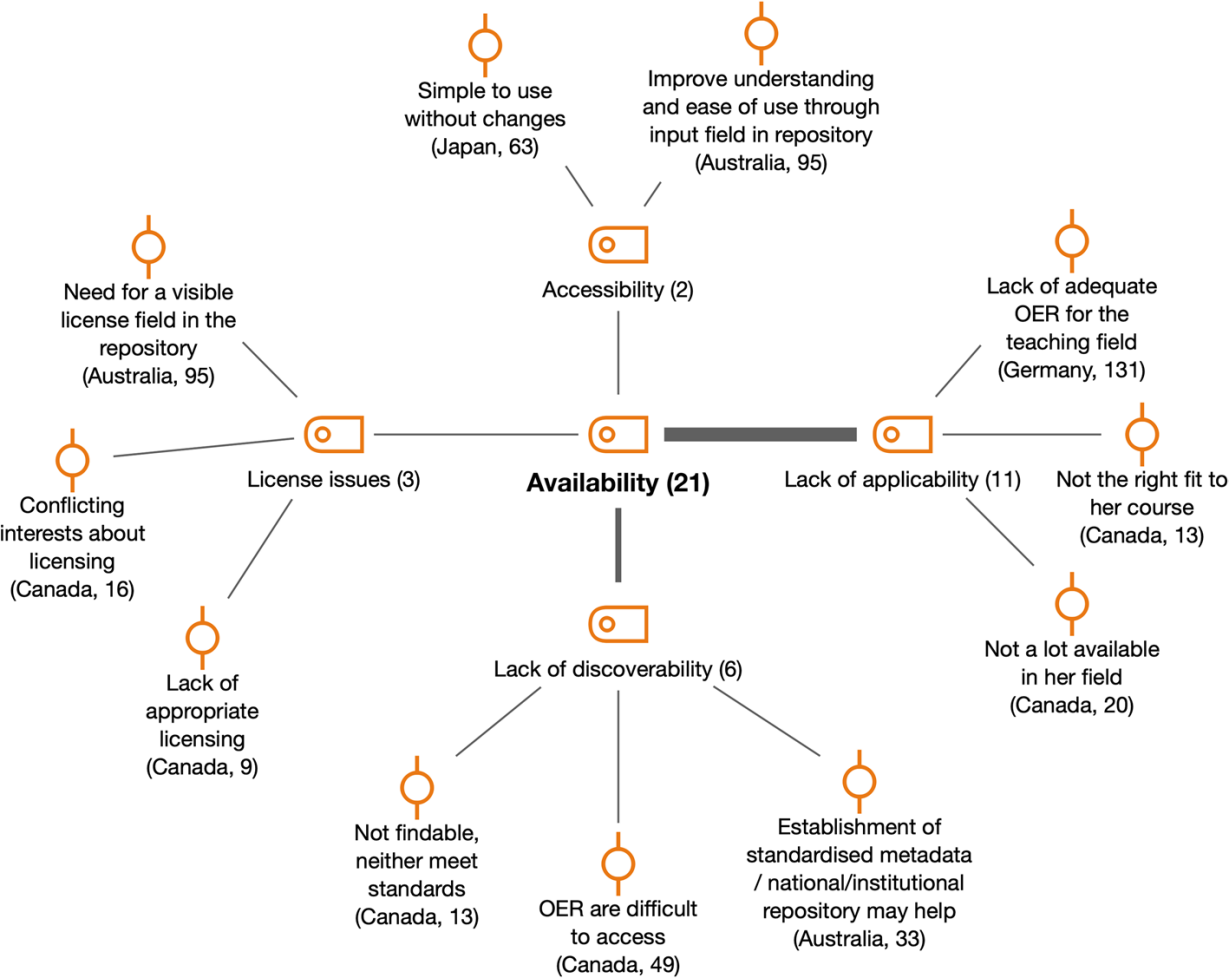
Subcodes and examples for availability (RQ1)

If we look at the OER Adoption Pyramid framework, the main category involved in OER Infrastructure is *Availability* as an additional challenge to interoperability (see Fig. 5).

Some of the elements included within the availability of OER have been also covered in previous literature. One survey participant in the Australian study stated that “unfortunately the repository does not have a visible license field which undermines our ability to support content in terms of infrastructure.” Also, our study found that a lack of discoverability of OER was a challenge in Australia and Canada; this was also identified in other studies in the Netherlands (Baas et al., 2019), Tanzania (Mtebe & Raisamo, 2014) and the USA (Belikov & Bodily, 2016).

### Research question 2: Awareness and perceptions of OER quality assurance (QA)

**Fig. 6:**
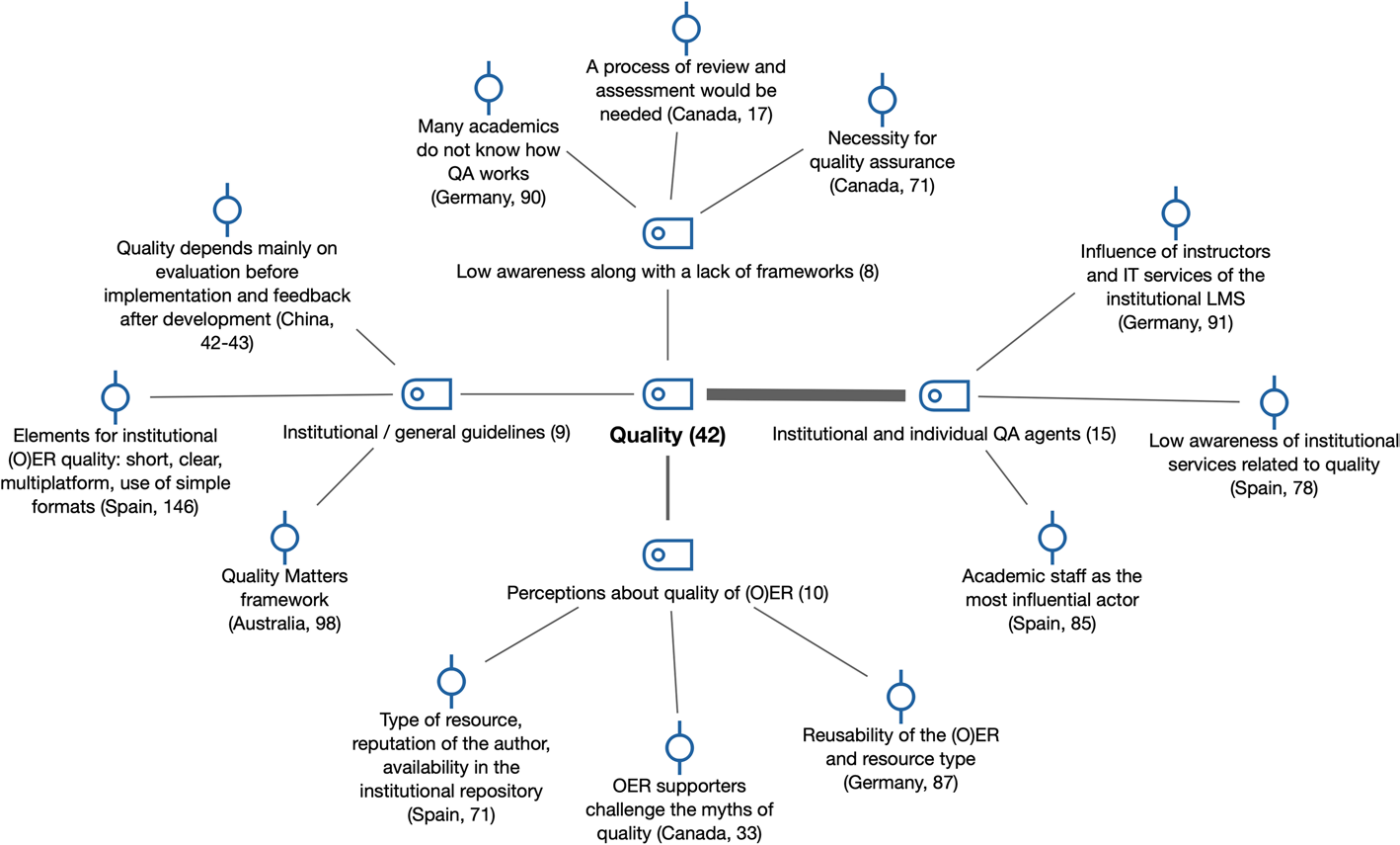
Subcodes and examples for quality (RQ2)

This research question explored how faculty defined the quality of OER, and how aware they were in terms of quality institutional measures and procedures, as well as of institutional agents for QA (see Fig. 6).

#### Faculty awareness and perceptions of OER quality

*Perceptions about the quality of OER* (*n* = 10) appeared in seven reports (Australia, Canada, Germany, South Africa, South Korea, Spain and Turkey). In many of the countries, these perceptions referred to a common prejudice against OER as being of low quality. For instance, in Turkey, openness and OER-related concepts were related to free sources with low quality. In South Africa, Madiba (2018) referred to lecturers’ concern about using OER by authors whose reputations are in doubt or not yet established. Interviewee E in Canada remarked that “OER supporters must challenge the ‘myths’ about their use and quality”. The poor quality of OER available and the concerns regarding the quality of content stored in OER repositories are common challenges found in the previous literature (Bates et al., 2007; Bossu et al., 2014; Mtebe & Raisamo, 2014).

In most of the countries studied, a *low awareness along with a lack of frameworks* regarding OER quality and their infrastructure (*n* = 8) was highlighted. For example, a survey study in China with 172 participants from eight universities identified a lack of supervision to ensure that faculty members implemented online teaching and OER of a high quality (Xu, 2018). In South Korea, a challenge pointed out by Lee and Kim (2015) for the active adoption of OCW was the lack of mechanisms to ensure the quality of OCW. This issue is in line with the lack of awareness pointed out previously (Baas et al., 2019; Li & Li, 2012; Schuwer & Janssen, 2018), but it also links to insufficient institutional support (Bossu et al., 2014).

#### Institutional and individual QA agents

In this section, we address faculty awareness concerning institutional quality assurance (QA) agents involved in OER, as well as faculty involvement as QA agents in OER at the teaching and learning level (*institutional and individual QA agents*, *n* = 15).

In China, Xu’s survey (2018) showed that a low percentage of faculty members (30.2%) agreed with the statement that “the university has a teaching team for developing OER,” whereas 25% responded with “completely disagree.” In Australia, the library played a key role in OER development at the Queensland University of Technology, through an optional stage of QA (Stevens et al., 2017). In Spain and Germany, the awareness of institutional QA agents reported by participants in the survey was low. The influence of IT services for the institutional LMS was perceived by faculty members as relevant in Germany (40.8%), but between 30 and 45% of participants were unsure or did not provide an answer to the question. Many of the remaining participants agreed that academic staff that use OER are the most influential actors as far as defining OER quality, of OER metadata and of OER repositories in the universities concerned (Spain: 41.2%; Germany: 42.1%). For instance, one participant in the Spanish survey stated that “it is a self-publication, there are no mechanisms of evaluation or quality in the repository. The OCW project died, it was not followed up”. This faculty involvement and responsibility for OER quality were also present in other countries too (e.g., Japan, Turkey).

### Research question 3: Awareness of OER policies

**Fig. 7:**
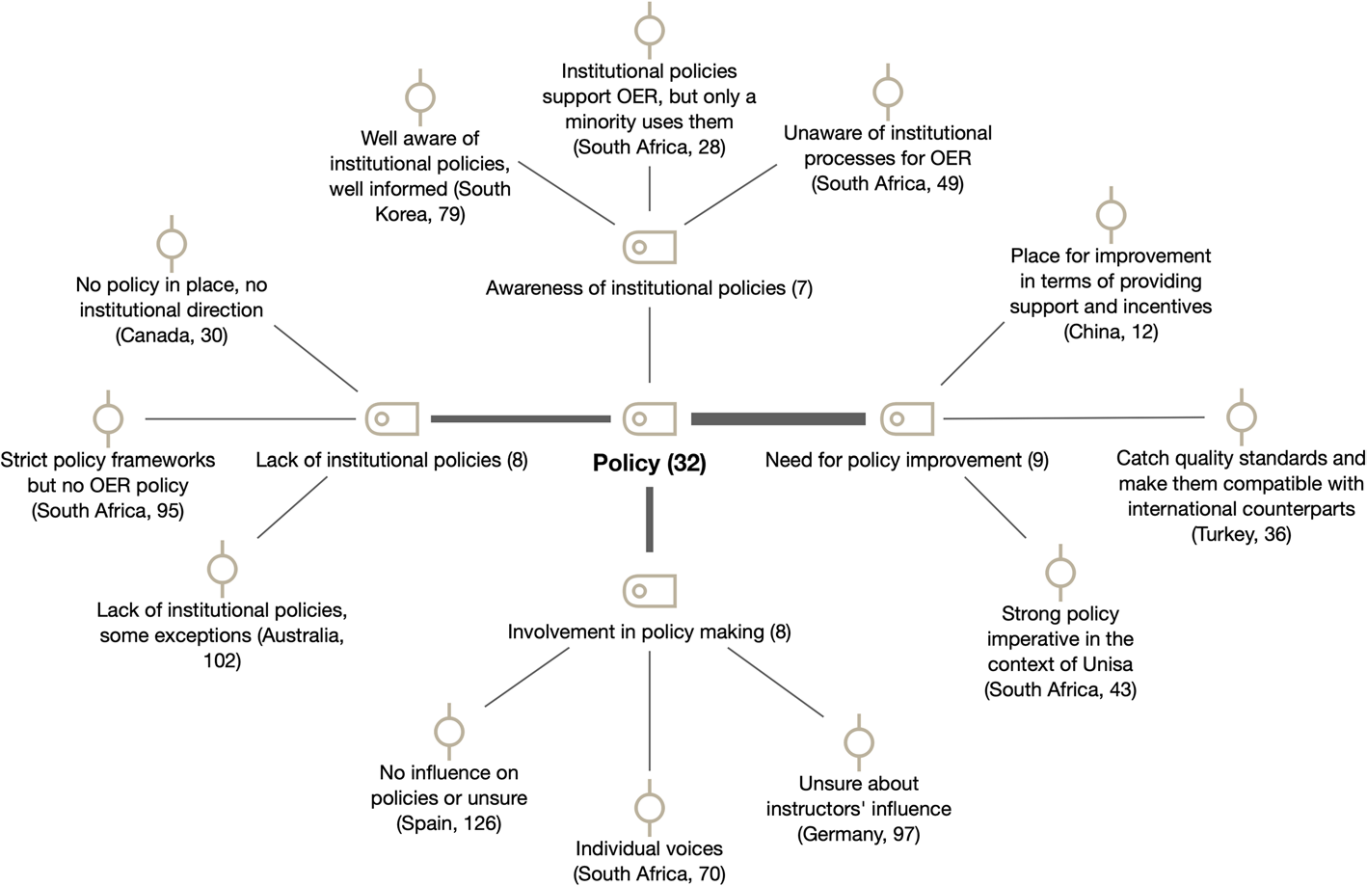
Subcodes and examples for policy (RQ3)

In this section, we focused on faculty involvement in policymaking and their awareness of institutional policies related to OER (see Fig. 7).

#### Specific institutional policies

A *lack of institutional policies* for OER (*n* = 8) was acknowledged in most of the countries. For instance, Canadian respondents E and H stated that there were no guiding institutional policies or direction in OER. In Australia, 64% of the survey participants indicated that explicit institutional OER policies or frameworks were non-existent in their institutions; a finding which is backed by a dearth of institutional policies noted in the literature (Open Education Licensing Project, 2016a). Previous studies also echo these findings in relation to the lack of institutional and departmental policies for the development and use of OER repositories as a barrier for the uptake of OER initiatives (Bates et al., 2007; Mtebe & Raisamo, 2014; Yuan et al., 2008).

Even in cases where some kind of OER policy was in place, the *need for policy improvement* (*n* = 9) was made explicit. A clear case of this is South Korea, despite its institutional emphasis on OER creation and utilization. Similarly, in the study survey by Wang and Wu (2013) at Peking University (China), 153 faculty members argued that more policies and mechanisms for motivating faculties to develop OER by protecting their intellectual properties were key to promoting OER. Cox and Trotter (2016) pointed out that a strong policy imperative would be crucial for faculty in the context of Unisa (the largest distance teaching university on the African continent, located in South Africa) to actively embrace OER. Also, de Oliveira Neto et al.’s (2017) findings in the Global South showed that OER-related policies did not seem to be relevant regarding OER use, but they do for OER creation. One of the participants in the survey study in Spain elaborated further on this topic regarding the situation in their institution:

There is a policy, but it will have to be improved and more widely disseminated. I do not believe that there is a lack of interest, on the contrary, but there is a lack of time and more measures in the direction taken so that it becomes part of the culture of the institution. Among these measures are […]: time, space, incentives, recognition...

In line with these findings, *faculty awareness of institutional policies* (*n* = 7) was low overall across the countries, with the same exception as before (South Korea). In Spain and Germany, most of the academics surveyed were unsure about the existence of institutional policies for specific study programs or for department/faculties (Spain, 67.4%; Germany, 56.6%). In addition, only around 20–25% of the Spanish and German participants surveyed stated that there was an explicit or implicit institutional policy or regulations concerning the use and/or creation of OER in their universities. Over half the participants were uncertain about this. In the study by Xu (2018) in China, only 33% of the participants were aware of relevant national policies, and only 37.2% knew about relevant university policies. These findings also echo previous works (e.g., Cox & Trotter, 2016).

#### Faculty involvement in policymaking

*Faculty involvement in policymaking* (*n* = 8) was present in some institutions but often reported as anecdotal cases. The exception was South Korea, where individual faculty members were regularly involved in policymaking via various committees and internal/external reviews. For instance, according to a field study at a Chinese university in Nanjing (Meng, 2018), faculty members were invited to attend seminars to give feedback on the policy for calculating their reduction in face-to-face teaching workload if they were using OER (online courses). As an Australian institutional case, the OER policy of the Queensland University of Technology was developed with the input of the University Copyright Officer, diverse units related to learning, teaching and IT, and various individual academics interested in OER (Open Education Licensing Project, 2016b).

When asked in the Australian survey which actors were involved in OER policymaking at their institutions, only 30% of participants provided some level of response. The most involved actors of OER policy mentioned were the libraries and, to a lesser degree, only “individual academics” or “individual/small group of educators who are OER champions.” The role of librarians was similarly exemplified by respondent E in Canada: by belonging to a provincial working group on OER, “she pushes her institution for change and for policy development.” Most of the surveyed academics in Spain and Germany were either not involved in the preparation of institutional OER policies (Spain, 36.3%; Germany, 19.7%) or uncertain about it (Spain, 54.6%; Germany, 57.9%). Regarding the possibilities of influencing explicit policies, both Spanish and German academics were mostly unsure (Spain, 57.8%; Germany, 64.5%).

While the role of (academic) librarians as change agents to promote open access within the institutions has started to be explored in previous literature (e.g., Mullen & Otto, 2014), it is noteworthy that specific librarian and faculty involvement in policymaking for OER and OER repositories seem to be still largely under researched.

### Research question 4: Promotion measures for faculty use of OER

Promotion of change at the microlevel was directly related to different parts of the OER Adoption Pyramid model; *permission, awareness, capacity and volition* in particular*.* In addition to this, individual volition had a clear and relevant extrinsic motivating factor: the presence or absence of incentives.

#### Faculty involvement in creating OER and advancing the infrastructures

To describe faculty involvement in OER, we need to acknowledge different elements that directly affect this involvement. The first of them is the factor *permission* (*n* = 8), which refers to institutional dispositions to which the academics are tied, particularly related to copyright issues and who owns OER developed by faculty members. For example, in Turkey, the current Law of Intellectual and Artistic Property Rights includes two articles to allow the use of OER for not-for-profit face-to-face educational processes, as long as the creators were cited; however, nothing was specified about open and distance learning. In South Africa, and particularly at Unisa, the institution owns all the intellectual property of work created by staff members, but at University of Cape Town academics are allowed to own it and, therefore, label it as OER (Cox & Trotter, 2016). In Canada, respondent A explained that part of the challenge in adopting OER is the issue of institutional ownership of OER created by faculty: “created material belongs to the institution, thus inhibiting some instructors from creating their own OER. Their contracts prevent them from seeking a CC license for their products.” Similarly, in many Australian HE institutions, OER “ownership is retained by the university—the lecturer must seek policy approval to release course materials outside of the institution” (Stagg & Partridge, 2019, p. 479). Intellectual property policies are one of the main problematic issues that arose in previous literature too and have been suggested as “the root cause of slow development in this field” (Bossu et al., 2014; Mtebe & Raisamo, 2014; Yuan et al., 2008, p. 16).

The second factor that affects faculty involvement in OER actions is *awareness* (*n* = 18), which refers to the degree of knowledge that faculty members have concerning OER and the philosophy behind openness. It was more common that faculty showed low rather than high awareness as shown by Chikuni et al. (2019) in South Africa, which also echoes previous work overall and in other countries (e.g., Baas et al., 2019; Li & Li, 2012; Schuwer & Janssen, 2018; Yuan et al., 2008). On the other hand, 83% of the participants in the Australian survey had previously heard of OER and, similarly, high levels of awareness of OER among a majority of the respondents from 4-year institutions were found out in a large-scale survey with educators from Japanese HE institutions (Shigeta et al., 2017), in contrast with studies from other contexts.

*Capacity* (*n* = 11) is the third relevant factor in faculty involvement with OER. Most of the reports highlighted some shortage of academics’ digital skills and emphasized the importance of institutional professional development support. For instance, in a survey of 119 academic staff members at Unisa (South Africa), Roberts (2016) found that the respondents’ perception of their own ability to be technically sound was very low and that training in this area was required. In Canada, according to respondent E, “a lack of technical skills (was an element that) impeded some (educators)”, which is reported in a previous study in the same geographical context: the need for educators to be better educated in OER-related skills, such as finding appropriate materials (Hayman, 2018). Interestingly, there was a comment from an interviewee from the Beijing National University Centre of Information and Network Technology who stated: “the most important factor that impacts on the (digital, including OER) implementation is IT literacy among leaders and administrative staff who are involved directly in digitalization work at the institutional level”. Similarly, a challenge pointed out by Lee and Kim (2015) for the active adoption of OCW in South Korea was the lack of digital competence at both faculty and institutional levels, which echoes previous research in China (Li & Li, 2012) and in Tanzania (Mtebe & Raisamo, 2014).

**Fig. 8:**
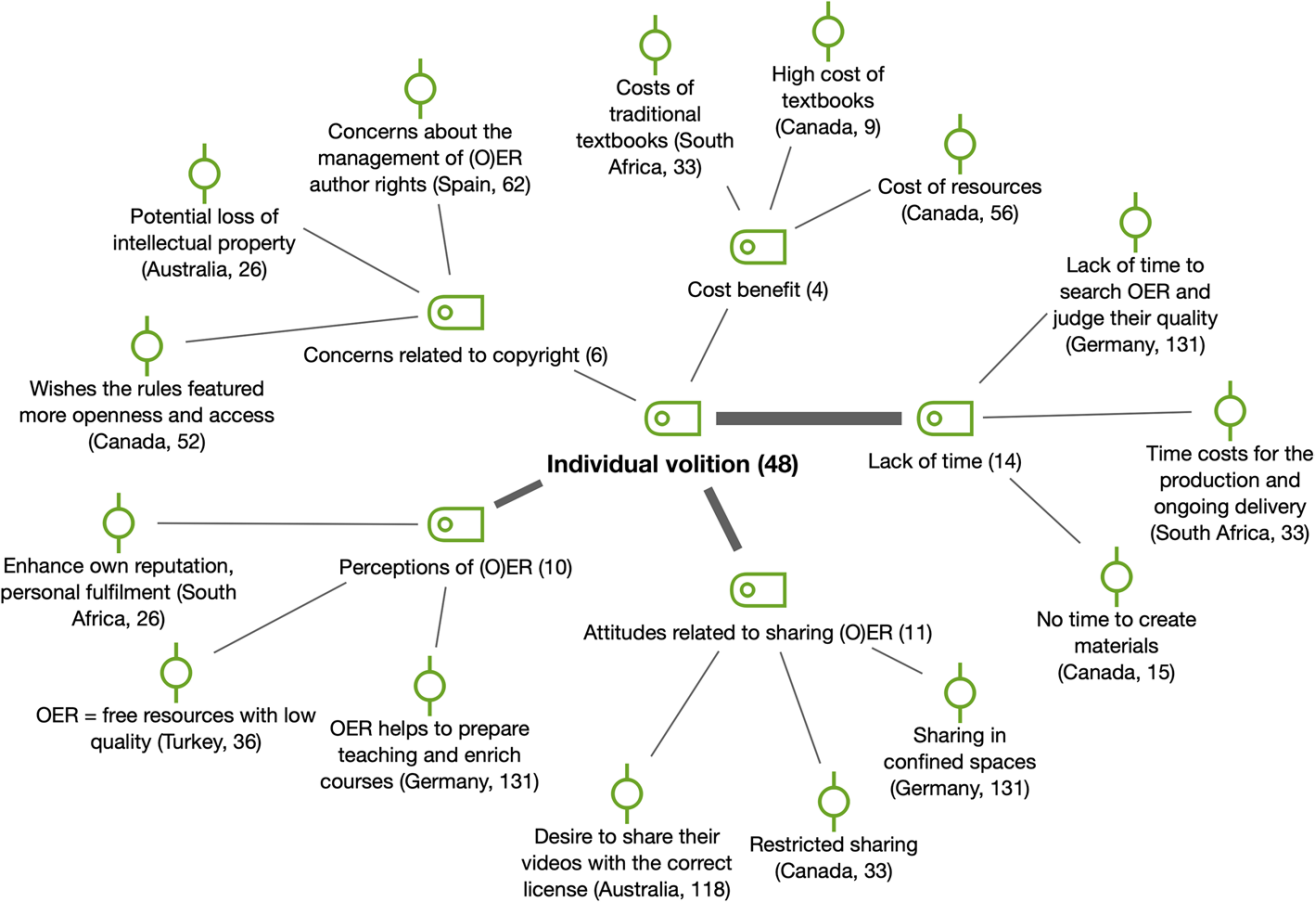
Subcodes and examples for individual volition (RQ4)

The last factor included here is *volition* and concerns the desire of faculty to create, use, adapt, remix and share OER when referred to *individual volition* (*n* = 48) (see Fig. 8), but there could be *social and institutional volition* too.

Many of the elements identified for *individual volition* appear in the literature related to the topic (Cox & Trotter, 2017). For example, student cost–benefit was an important factor in Canada and South Africa but was also present in the perceptions of US faculty members found in the previous literature (Belikov & Bodily, 2016); all of them are countries where OER, and in particular textbooks, are usually expensive. In these countries, but also in Australia, open textbook initiatives have flourished (Stagg & Partridge, 2019). Time restraints were a factor expressed in many of the reports, also supported by the literature (e.g., Baas et al., 2019; Bates et al., 2007; Cox & Trotter, 2017; Yuan et al., 2008).

Concerning *social volition* (*n* = 11), we refer to the social environment of the faculty members (department, other faculty members, colleagues) and how interested/resistant they are to be involved in OER processes, but also the influence that this exerts on individual volition through modeling or social desirability. An element that stood out in the reports of Canada and China was the presence of OER forerunners as inspiration for colleagues at their institutions or even at a broader level. In Japan, social influence from peers was highlighted as more important than improving performance in regard to adopting OER, which emphasizes the relevance of culture (Jung & Lee, 2020), in contrast with a study in the context of Tanzania (Mtebe & Raisamo, 2014). On the other hand, Canadian respondents B and E reported that faculty members in their institutions were reluctant to use or trust repositories and OER.

**Fig. 9:**
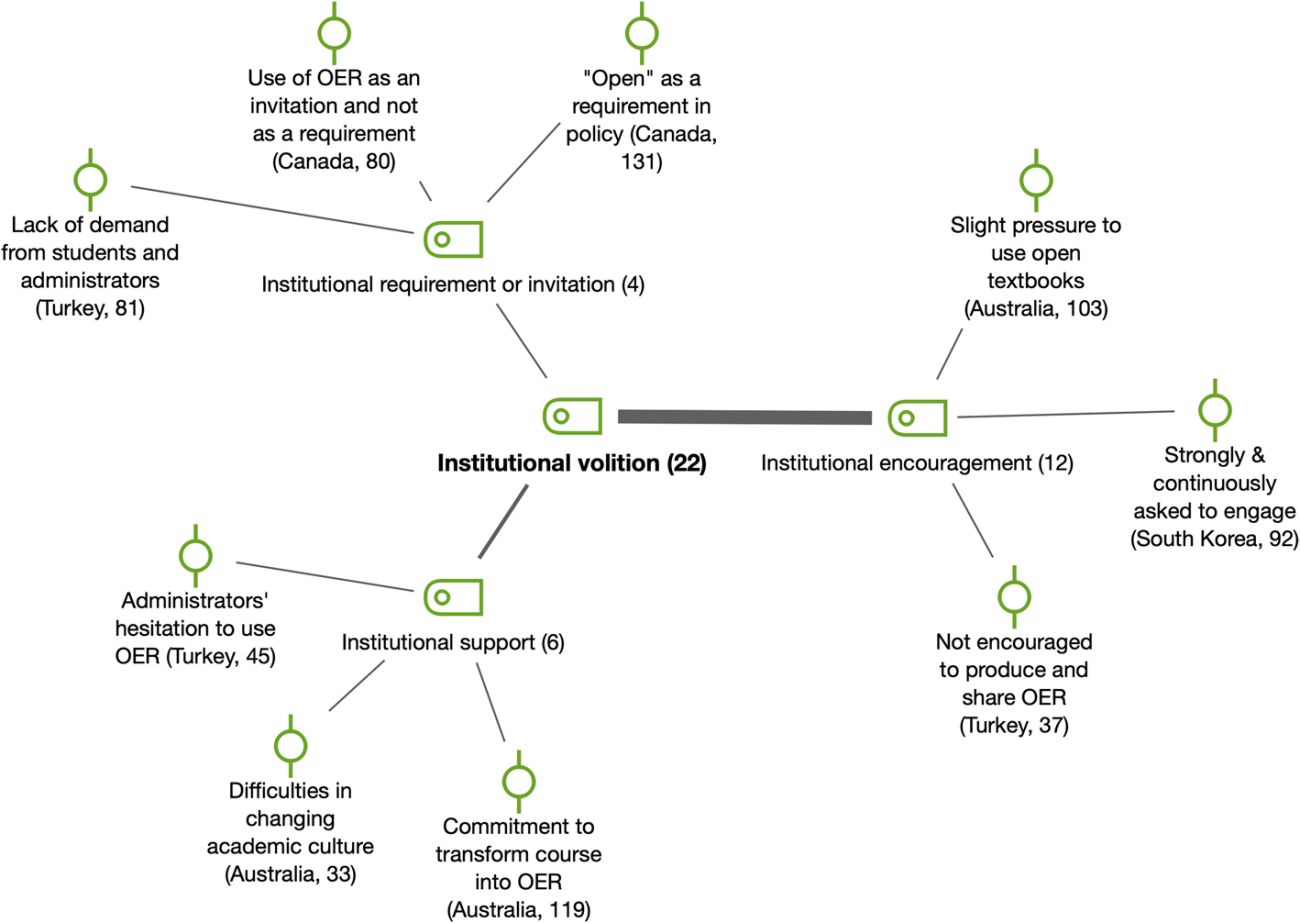
Subcodes and examples for institutional volition (RQ4)

*Institutional volition* (*n* = 22) is another factor related to the development and adoption of OER, and it refers to the interest of the institution to push OER forward (see Fig. 9), e.g., through institutional encouragement, commitment or requirement. For example, a survey participant in the Australian study stated that “unofficially, slight pressure is being applied to lecturers at a very low level to encourage them to consider open textbooks as a cost-reduction measure for students”. The influence of institutional volition is also a relevant element considered in the literature (e.g., Cox & Trotter, 2016).

#### Support for faculty in OER creation

Faculty were supported in creating, sharing, using and remixing OER and using repositories by two main elements at this level: institutional professional development support (addressed to improve *capacity*) and the use of incentives (addressed to increase *individual volition*). Both elements have been recognized as key elements for individual OER promotion by previous works within this research area (Belikov & Bodily, 2016; Cox & Trotter, 2016; Murphy, 2013).

**Fig. 10:**
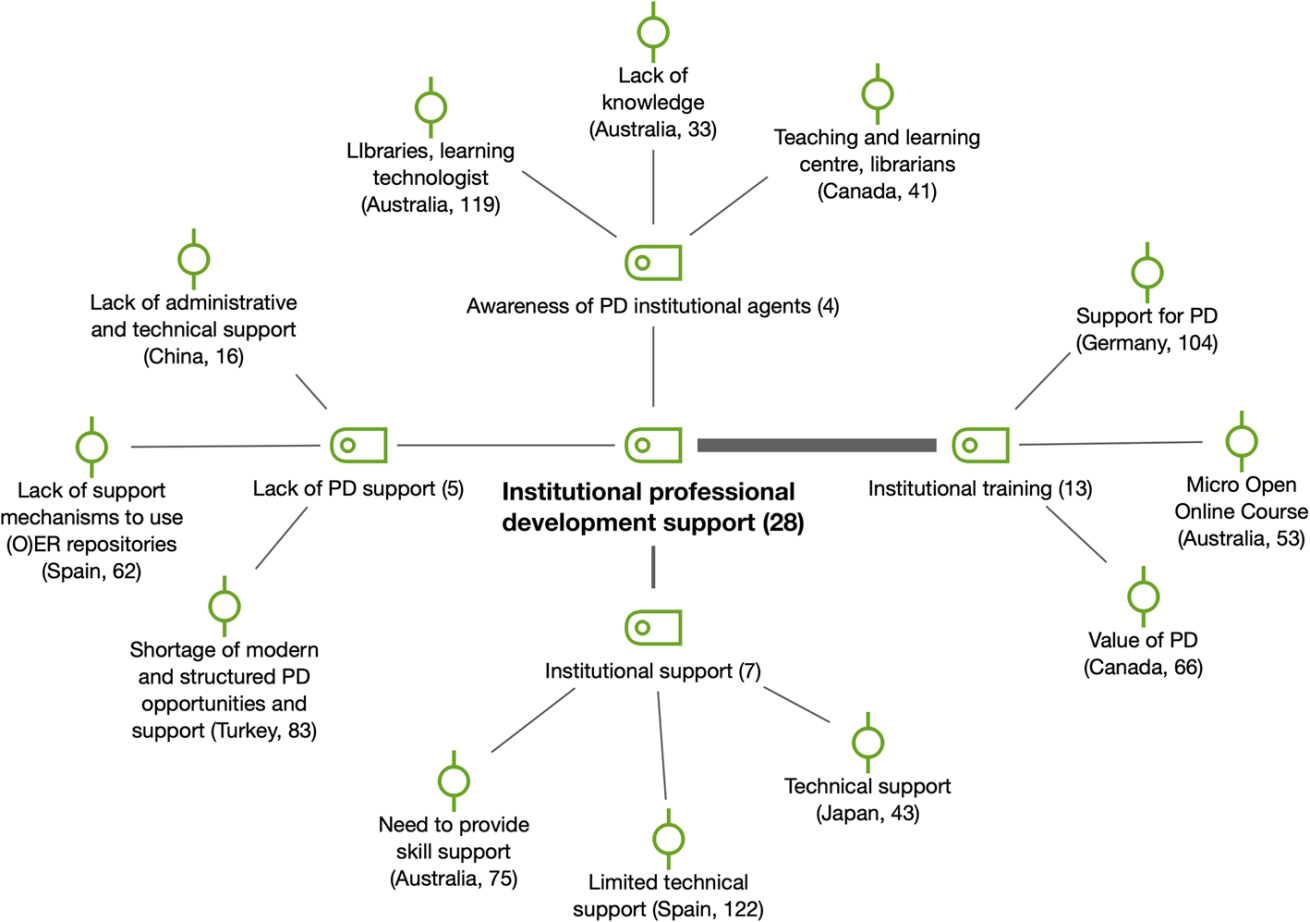
Subcodes and examples for institutional professional development support (RQ4)

In terms of *institutional professional development support* (*n* = 28), most of the reports mentioned different forms of this support and training. We considered institutional training and support as two different professional development (PD) aspects as well as faculty awareness of PD institutional agents (see Fig. 10). For example, a participant in the Spanish survey stated that “in some cases, support has been offered for creating knowledge pills and for creating resources to incorporate in MOOCs. Some initiatives are supported by the teaching innovation program of the university”.

**Fig. 11:**
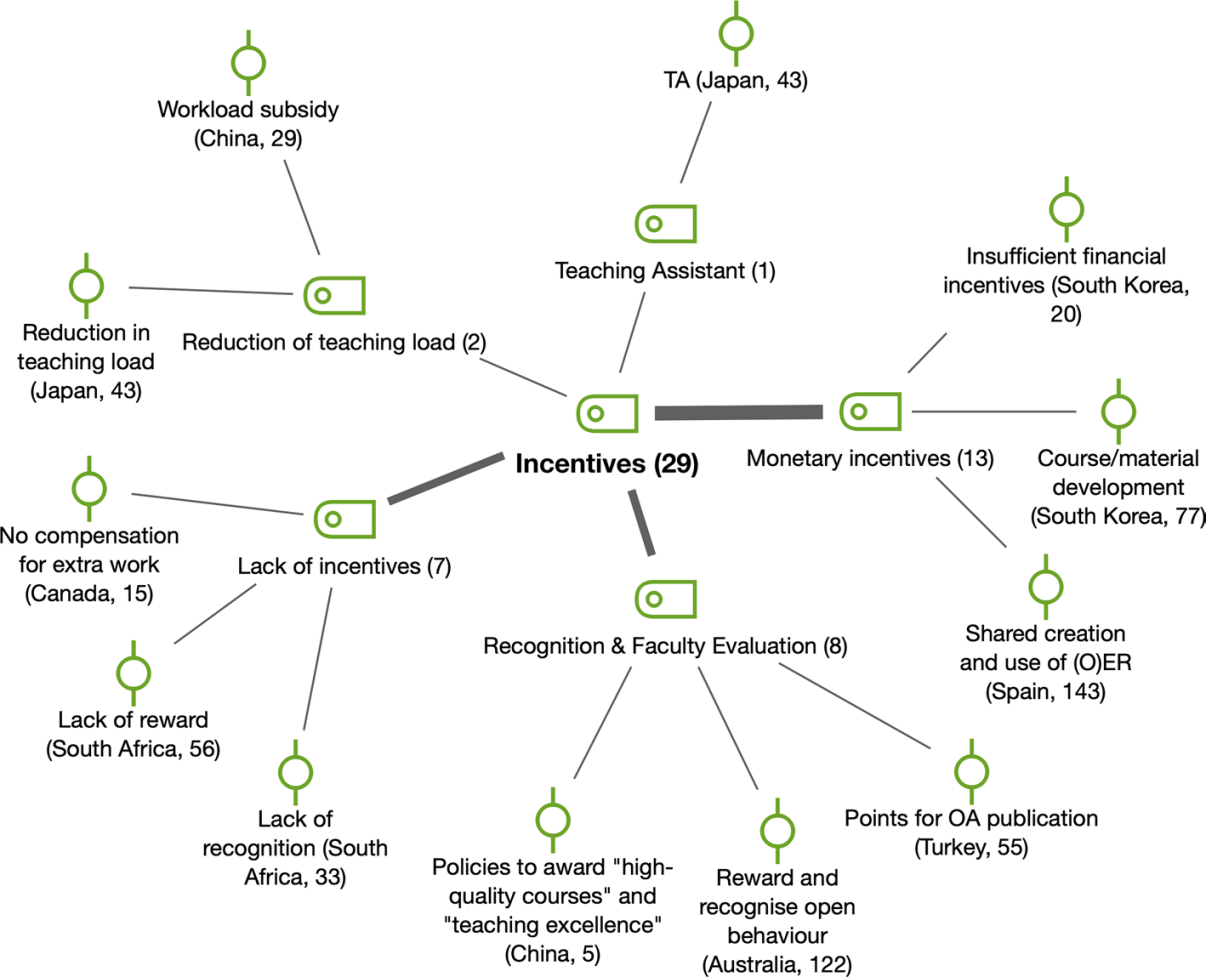
Subcodes and examples for incentives (RQ4)

*Incentives* (*n* = 29) were important measures to support change to OER at the individual level (see Fig. 11). Diverse kinds of incentives were mentioned in the reports: the assignment of teaching assistants, measures for recognition and faculty evaluation, the reduction in teaching load, as well as monetary incentives. For instance, an interview participant from China that had previously held a managerial role at Beijing Open University stated that “(the university) has policies to award ‘high-quality-courses’ and ‘teaching excellence’ to faculty members who prove themselves able to create high-quality educational resources or who demonstrate excellent instructional designs in their courses every year”. However, *lack of incentives* (*n* = 7), also present in the literature (e.g., Yuan et al., 2008) and especially related to economic compensation, was remarkable in countries such as Canada, South Africa and Spain, as the following quotation from a Spanish survey participant describes:

They are valued but, in short, they are made by teaching vocation and teaching conviction. They are not compensated financially, and it is very time consuming (to create them). It only produces personal and teacher satisfaction, in no case economic satisfaction, at least not at present.

#### OER sharing, integration and remixing by faculty

*OER practices* (*n* = 24) by faculty members in the reports show that OER uptake is overall in its infancy, which could also be explained by factors identified in the previous sections. This would also correspond to what the Open Educational Quality (OPAL) initiative of the Open Educational Practices (OEP) matrix presents as “early stages/awareness” (Conole, 2012).

Although many of the reports mentioned OER use and development, much less space was devoted to describing practices beyond these activities (e.g., sharing, remixing, integrating) and some challenges were highlighted. For instance, the continuous use of OER without an opportunity for revisions or updates was identified among the barriers to South Korean faculty involvement in the creation of OER (Lim et al., 2017). De Hart et al. (2015), in their study with Unisa staff (South Africa), found that “activities relating to the use of OER (accessing, redistributing and re-using) are far more frequent than activities relating to contributing to OER (revision, remixing, developing)” (p. 32). Similarly, the survey studies in Spain and Germany showed that many faculty members did not use OER from repositories (68% and 54%), search for OER in them (48% and 55.3%), and neither published in them (48% and 43.4%) nor in non-institutional repositories (68.8% and 86.8%). These findings echo Reed’s (2012) study in the UK with regard to formal, large-scale sharing of educational materials in specific OER repositories.

In Canada, concrete OER practices were shown by respondents H, F and C. For instance, respondent C had been an avid OER creator for several years and co-created together with her students a textbook with eCampus Ontario which was then published via PressBooks. Furthermore, she “would rather invest the time in adapting materials to her own needs than re-invent the wheel” and “share(s) relevant material (with her colleagues), ‘the good stuff’, in its original format, often by email,” unofficially. This finding related to that of Baas et al. (2019) in the Netherlands, as well as Reed (2012) and Rolfe (2012) in the UK, where faculty frequently shared resources informally, however, the current findings differ when it comes to willingness to adapt OER in Baas et al. (2019).

Along the same lines, despite high levels of OER awareness and knowledge by participants, the majority of faculty members in Australian HE involved in the study by Bossu et al. (2014) had rarely or never used, developed, and/or re-purposed OER. In the survey, a participant mentioned that there was a push to “tag everything as being an OER upon completion” but that there “is very small uptake” as regards use or storage of OER in institutional repositories.

## Conclusion

This study contributed to the literature by providing new insights into the factors that influence OER adoption by faculty in various countries and by emphasizing the importance of factors that have been previously identified in the literature. This study also offers a revision of the OER Adoption Pyramid as an analytical framework to consider further elements that were investigated in the project at higher levels (macro and meso level perspectives).

Key findings of the study are several. In RQ1, we identified commonalities in the use of OER (i.e., slide presentations and videos) and their repositories (low awareness and use) by faculty members in various countries. External OER platforms such as YouTube were well known and used as sources. Faculty perceptions referred to the diverse challenges concerning OER infrastructure, in particular, the availability of OER.

In RQ2, we explored the awareness of faculty members of institutional procedures related to OER QA and QA agents across countries. A low awareness along with a lack of frameworks was highlighted, as well as the importance of faculty members as agents of QA at the micro-level. Similar findings were reported in RQ3 regarding faculty awareness of institutional OER policies and involvement in policymaking: a lack of institutional policies along with a low awareness of them was common. Even in the exceptional cases where this situation was not the case, a need for policy improvement was made clear.

Finally, in RQ4, we identified the need for encouragement measures to be implemented by HE institutions, in order to motivate faculty to use OER. Diverse kinds of incentives and institutional professional development and support were highlighted, but it was also made explicit that individual, social and institutional volition influence the actual individual decision to use OER. Within OER practices, the fact that the emphasis made by institutions (when existing) was on using and creating OER, but less often on co-creating, remixing and sharing them, was remarkable. Notwithstanding the limitations of the study in terms of data collection, the findings offer possibilities of reflection and action for HE institutions and administrators across countries and invite faculty to learn from the experiences of these international HE academics.

Overall, despite various calls for OER and OEP on the political agenda in many countries, this international multiple case study has shown that the current state of OER awareness and adoption among faculty members is (still) disappointing and leaves room for improvement and development. Concrete implications of the study for HE institutions to foster faculty use of OER could point toward the improvement in the availability of OER in institutional repositories and the development of measures for dissemination and faculty involvement in OER policy and QA. HE institutions may also consider effective monetary and recognition incentives, which might include not only a reduction in workload but also the development of professional development training and support that is directly targeted at OER copyright issues and strategies for properly finding, remixing, sharing and co-creating OER beyond simple OER individual use and/or creation. We believe that these institutional measures may address most of the factors identified for individual volition and encourage faculty to use, remix, share and publish OER at the micro-level.

Future research may consider co-design processes for institutional OER promotion and adoption between faculty and administrators. The long-term impact of the emergency remote teaching during the COVID-19 pandemic on faculty interest and practices with OER at the microlevel may also lead to rich insight and provide further guidance for policy, creation and implementation going forward.

## Data Availability

Not applicable.

## Abbreviations

HE: Higher education
OEP: Open educational practices
OER: Open educational resources
PD: Professional development
QA: Quality assurance
RQ: Research question
